# Home gardening improves dietary diversity, a cluster‐randomized controlled trial among Tanzanian women

**DOI:** 10.1111/mcn.13096

**Published:** 2020-11-26

**Authors:** Mia M. Blakstad, Dominic Mosha, Alexandra L. Bellows, Chelsey R. Canavan, Jarvis T. Chen, Killian Mlalama, Ramadhani A. Noor, Joyce Kinabo, Honorati Masanja, Wafaie W. Fawzi

**Affiliations:** ^1^ Department of Global Health and Population Harvard T.H. Chan School of Public Health Boston Massachusetts USA; ^2^ Ifakara Health Institute Dar es Salaam Tanzania; ^3^ Department of Social and Behavioral Sciences Harvard T. H. Chan School of Public Health Boston Massachusetts USA; ^4^ Department of Nutrition Harvard T. H. Chan School of Public Health Boston Massachusetts USA; ^5^ Department of Food Science Technology, Nutrition and Consumer Sciences Sokoine University of Agriculture Morogoro Tanzania; ^6^ Department of Epidemiology Harvard T. H. Chan School of Public Health Boston Massachusetts USA

**Keywords:** community based, food and nutrient intake, food security, low income, maternal nutrition, micronutrients, nutrition‐sensitive agriculture, homestead food production, community health workers, dietary diversity, leafy greens

## Abstract

Homestead food production (HFP) programmes improve the availability of vegetables by providing training in growing nutrient‐dense crops. In rural Tanzania, most foods consumed are carbohydrate‐rich staples with low micronutrient concentrations. This cluster‐randomized controlled trial investigated whether women growing home gardens have higher dietary diversity, household food security or probability of consuming nutrient‐rich food groups than women in a control group. We enrolled 1,006 women of reproductive age in 10 villages in Pwani Region in eastern Tanzania, split between intervention (INT) and control (CON) groups. INT received (a) agricultural training and inputs to promote HFP and dietary diversity and (b) nutrition and public health counselling from agricultural extension workers and community health workers. CON received standard services provided by agriculture and health workers. Results were analysed using linear regression models with propensity weighting adjusting for individual‐level confounders and differential loss to follow up. Women in INT consumed 0.50 (95% CI [0.20, 0.80], *p* = 0.001) more food groups per day than women in CON. Women in INT were also 14 percentage points (95% CI [6, 22], *p* = 0.001) more likely to consume at least five food groups per day, and INT households were 6 percentage points (95% CI [−13, 0], *p* = 0.059) less likely to experience moderate‐to‐severe food insecurity compared with CON. This home gardening intervention had positive effects on diet quality and food security after 1 year. Future research should explore whether impact is sustained over time as well as the effects of home garden interventions on additional measures of nutritional status.

**Key messages**

- Training women in homestead gardening and nutrition concepts improves their dietary diversity and consumption of nutrient‐dense foods but not household food security.
- This is the first randomized trial to find statistically significant impacts of a home gardening programme on women's dietary diversity
- The results were achieved over a relatively short period of 12 months, and future research should examine whether the effects are stronger, smaller or maintained in the long run.

**LIST OF ABBREVIATIONS**
AEW: agricultural extension worker
BMI: body mass index
CHW: community health worker
CON: control group
FFS: farmer field schools
HFIAS: Household Food Insecurity Assessment Scale
HFP: homestead food production
INT: intervention group
LEW: livestock extension worker
MDD‐W: minimum dietary diversity for women
MFI: moderate food insecurity
RD: risk difference
SFI: severe food insecurity

## 1 INTRODUCTION

Malnutrition remains a major public health problem in rural Tanzania and other countries in sub‐Saharan Africa (Food and Agriculture Organization [FAO], The International Fund for Agricultural Development [IFAD], United Nations Children's Fund [UNICEF], World Food Programme [WFP], & World Health organization [WHO], 2017). In these settings, a large percentage of the population consumes mostly carbohydrate‐dense staples such as maize, rice, cassava and potatoes that have low concentrations of essential micronutrients needed to maintain health (Bellows et al., 2019; Huang et al., 2018; Lander et al., 2019). Micronutrient‐dense foods such as vegetables and animal products are often more expensive and perishable than staple grains and therefore less affordable, especially for low‐income households (Masters et al., 2018). Nutrient‐dense foods can also be difficult to access in areas with limited access to markets or for families with limited financial means. Diverse diets are beneficial for improving nutritional status, cardiovascular health, pregnancy outcomes and vision (Abriha, Yesuf and Wassie, 2014; Li et al., 2015; Mwanri, Kinabo, Ramaiya and Feskens, 2015; Narmaki et al., 2015; Shiraseb et al., 2016). Dietary diversity scores have been developed as an indicator of the micronutrient adequacy of the diet (Arimond et al., 2010; Arsenault et al., 2013; Becquey et al., 2010; FAO & FH360, 2016; Women's Dietary Diversity Project Study Group, 2017).

Homestead food production (HFP) programmes provide participants with tools and knowledge for growing local, nutrient‐dense crops in home gardens thereby improving access and availability of vegetables for the household. Additionally, HFP can improve health through financial gains from vegetable sales, thereby generating income that can be used to access health services or to buy food in markets (Olney et al., 2016). According to a systematic review, integrated agricultural interventions show promise for improving women's nutrition and health (Girard, Self, McAuliffe and Olude, 2012). However, the majority of published literature is based on heterogeneous, quasi‐experimental studies with limitations to internal validity and other methodological drawbacks. The review called for additional research that is methodologically robust to make causal claims. In a review of more recent evidence, Ruel and colleagues found that only three trials employed robust study designs to evaluate the effect of HFP on nutritional status and indicators (Ruel, Quisumbing and Balagamwala, 2018). The effects on nutritional status were mixed: authors found reductions in the proportion of women who were underweight and in the proportion of women and children with anaemia, but no impact on child anthropometry in Nepal; reductions in wasting but not child stunting or on infant and young child feeding practices (IYCFs) in Zambia; and reductions in the proportion of women who were underweight but nonsignificant on women's dietary diversity in Burkina Faso (Kumar et al., 2018; Olney et al., 2016; Osei et al., 2017). Only the trial in Burkina Faso evaluated the impact of HFP on dietary diversity and found significant effects on individual food group consumption but not on overall women's dietary diversity or quality.

We conducted a cluster‐randomized trial in Rufiji District, Pwani Region, Tanzania, to examine the effect of HFP on dietary diversity and quality. We assessed improvements between baseline and 12 months of follow up in both dietary diversity and individual intakes of food groups among women. We hypothesized that women in intervention villages (INT) would be better off than women in control villages (CON) in terms of women's dietary diversity, household food security, and the probability of consuming (a) five or more food groups per day, (b) dark green vitamin A‐rich vegetables and (c) other vitamin A‐rich vegetables and fruits.

## 2 METHODS

This analysis considers data from a pair‐matched cluster‐randomized trial (ClinicalTrials.gov). The study design and population have been described in detail elsewhere (Mosha et al., 2018). Briefly, the study was implemented in Rufiji District, Pwani Region, Tanzania. Ten villages were randomly sampled from the Rufiji Health and Demographic Surveillance System (HDSS), a database that provided demographic and descriptive data on households in the study area (Mwageni et al., n.d.). The villages were matched into five pairs based on location, proximity to water source that could be used for irrigation (for instance, river, well or running tap water) and population size. Villages in each pair were randomly assigned to INT or CON. Randomization was done by colleagues with no prior knowledge of the villages. Households within each village were selected based on their eligibility and were approached by field workers for enrolment and informed consent.

Households that met the following eligibility criteria were recruited into the study: (a) had a woman between 18 to 49 years of age at time of recruitment and at least one child younger than 36 months of age, (b) household had access to a plot of land where vegetables can be grown and (c) provided informed consent. Across INT and CON, a total of 1,006 women were recruited into the study between August and October 2016, and a follow‐up assessment was conducted at 12 months postintervention initiation. For this pilot trial, we aimed to assess the feasibility and preliminary effectiveness of the intervention on dietary diversity and have not done formal power calculations in establishing sample size. The sample size of 1,000 households was predetermined by what was both logistically feasible for a time period of 1 year and also sufficient for pilot implementation, but no formal sample size calculation was performed a priori (for more details, please see Mosha et al., 2018).

### 2.1 Programme description

The intervention included two main components: (a) agricultural training and inputs to promote HFP and (b) nutrition and public health counselling to improve diet and health‐related behaviours. CON received the standard of care for the area, where agricultural extension services are offered in a nonstandardized way and community health services had not been established.

Agricultural training consisted of 15 main topics: (a) overview of homestead gardening, (b) fertilizer management, (c) different types of fertilizer, (d) agronomical practices and irrigation, (e) controlling harmful insects and vegetable diseases, (f) pests and pest management, (g) harmful effects of pesticides, (h) irrigation support, (i) farm processes, (j) crop harvesting, (k) composting, (l) marketing of vegetables, (m) nursery preparation, (n) raised bed preparation and (o) transplanting. Participating households received three or four types of seeds out of six local crop varieties (African eggplant, amaranth, spinach, tomato, okra and Chinese cabbage, all from the Kibo Seeds Company) at least three times during the study period. Seed types were selected based on local climatic and production conditions and local preferences. Households also received urea and cow manure fertilizer and watering cans.

The nutrition and health counselling for behaviour change consisted of 15 main topics: (a) food and water safety and hygiene, (b) food preparation and storage, (c) immunization, (d) handwashing, (e) key nutrition terms and importance of human nutrition, (f) maintaining a balanced diet, (g) physical activity, (h) food equivalents to meet energy needs, (i) causes and consequences of malnutrition, (j) breastfeeding, (k) complementary feeding, (l) advantages of vitamin A supplementation and deworming, (m) maternal and child health seeking behaviour, (n) importance of anthropometric measures and (o) importance of consuming iodized salt.

The intervention engaged the existing community workforce of agricultural extension workers (AEWs), livestock extension workers (LEWs) and community health workers (CHWs) to deliver agriculture training and behaviour change messages to participants. Messages were delivered through two main mechanisms: households received visits from either AEWs/LEWs or CHWs every 2 weeks on a rotating basis. AEWs, LEWs and CHWs all received training on all topics for the intervention according to the training manual, including agricultural practices, basic health messages (such as water, sanitation and hygiene) and nutrition (including the importance of dietary diversity). All workers were trained on nutrition messaging so that while CHWs focused on health and nutrition messages, AEWs and LEWs were able to reinforce these messages. Additionally, approximately every 2 weeks, farmer field schools (FFS) were held in collaboration by AEWs, LEWs and CHWs. The FFSs were held at the garden of a participating household, with a typical attendance of 10–15 programme participants from the nearest hamlet. During the FFSs, messages from the household visits were reinforced, benefits of improved agricultural practices were demonstrated (with nonparticipant households welcome to attend), and community knowledge about local availability of nutritious crops was shared. The field schools also served as a forum for collaboration and discussion and as a platform for women's empowerment as successful model farmers shared their experience and taught their peers best practices for home gardening.

The study manager continuously monitored the AEWs and CHWs to ensure routine delivery of the intervention components, both at household visits and FFS sessions. Each week, two participants were randomly drawn from each intervention village to be visited by the field manager for monitoring purposes.

### 2.2 Data collection

Data were collected at baseline and after 1 year. Surveys were administered on electronic tablets by trained interviewers. The survey questionnaires were developed by the research team and included modules on household socio‐economic status, food frequency intake, HFP and food security. Each household was assigned a composite wealth score derived from household assets (roof type, whether roof leaks, floor type, electricity, couch, television and bike ownership) using principal components analysis (Filmer and Pritchett, 2001).

### 2.3 Study outcomes

Outcomes for this analysis include dietary diversity and food security. The primary outcome for the trial was dietary diversity, prespecified in the ClinicalTrials.gov registration, while the secondary outcome food security was added post hoc. Dietary diversity was measured as the number of food groups consumed out of 10 using a locally adapted food frequency questionnaire (FFQ) that has been tested for validity (Zack et al., 2018) and used in previous trials (Bliznashka et al., 2020; Gerber et al., 2020). Participants were asked the average frequency of consumption of a given food item over the past 30 days using options ‘0 times in a month, ‘1–3 times per month’, ‘1 time per week’, ‘2–4 times per week’, ‘5–6 times per week’, ‘1 time per day’, ‘2–3 times per day’, ‘4–5 times per day’ and ‘6+ times per day’. The responses to these questions were used to calculate daily frequencies of consumption for each item. Frequency of consumption of each food item was aggregated into consumption of the following 10 food groups according to FAO guidelines: starchy staples (e.g. maize, bread and rice), flesh foods (e.g. beef, fish and chicken), vitamin A‐rich dark green vegetables (e.g. spinach, Chinese cabbage and sweet potato leaves), other vegetables (e.g. lettuce, eggplant and cucumber), other fruits (e.g. banana, guava and watermelon), other vitamin A‐rich vegetables and fruits (e.g. mango, papaya and tomato), dairy products (e.g. milk and ice cream), beans and peas (e.g. kidney beans, chickpeas and green peas), eggs, and nuts and seeds (e.g. Bambara nuts and ground nuts). Food items included in each category are detailed in the supporting information. A participant was considered to consume a given food group if the sum of daily frequencies for all foods in that group equalled or exceeded one. The dietary diversity score was then defined as the number of food groups consumed out of 10. Minimum dietary diversity for women (MDD‐W) was defined as a participant consuming five or more food groups per day (FAO & FH360, 2016).

Food security, a secondary outcome, was measured using the Household Food Insecurity Assessment Scale (HFIAS), a measure of food insecurity produced by Coates et al. (2007). The questionnaire operates under the assumption that levels of food access and insecurity produce predictable responses that can be expressed and quantified in a score. Such responses include quantity, quality and intake of food; perceived uncertainty or anxiety for food situations; and associated consequences. The HFIAS scale was also categorized to no food insecurity or mild, moderate and severe food insecurity (SFI) (Coates et al., 2007).

### 2.4 Statistical analysis

We report descriptive statistics of the study population using means and frequencies in Table 1. Statistical analysis was conducted using Stata 15.1 (StataCorp LP). Data were analysed based on the intent‐to‐treat principle. Our analytic strategy is threefold: First, we present ‘unadjusted’ estimates from our pair‐matched trial design, which conditions exclusively on village‐level characteristics captured by the three criteria used for matching villages (location, proximity to water and population size) per the methods recommended by Imai et al. (2009). Second, we present pair‐matched results weighted by the probability (propensity) of treatment conditional on measured individual‐level confounders. These methods have been comprehensively described by Hernán and Robins (2020). We included inverse probability of treatment weights because we felt that there was not sufficient balance on individual covariates after the pair‐matched cluster randomization, and treatment weighting allows for adjustment by measured individual‐level confounders. Third, we present results weighted by propensity of both treatment and censoring. We included weights for censoring because we detected a differential loss to follow up between INT and CON. Of the 1,006 (504 INT and 502 CON) households enrolled in the study at baseline, 455 in INT and 421 in CON were reached at 12 months (2017). The sample was subjected to a 12.9% loss to overall follow up: 9.7% in INT and 16.1% in CON. Reported reasons for loss to follow‐up were out‐migration (45%), travel during data collection (30%), married out of village (13%), divorced out of village (6%), refusal (4%) or other reasons (5%) (see CONSORT chart, Figure S1).

**TABLE 1:** Baseline characteristics of women in CON and INT villages

Variable	CON (*N* = 422)	INT (*N* = 454)	*N*
Woman age at 12 months follow up (years), mean ± SD	31.6 ± 7.5	31.6 ± 8.3	874
Wealth quintile score *n* (%)			994
First (lowest)	73 (14.3)	120 (23.8)	
Second	101 (20.1)	80 (15.9)	
Third	123 (24.5)	109 (21.6)	
Fourth	93 (18.5)	97 (19.3)	
Fifth (highest)	108 (21.5)	91 (18.1)	
Education *n* (%)			1,005
No education	155 (30.9)	173 (34.3)	
Primary	288 (57.5)	290 (57.5)	
Secondary	56 (11.2)	35 (6.9)	
Higher education (high school or higher)	2 (0.4)	6 (1.2)	
Type of employment *n* (%)			992
No income generating activity	87 (17.4)	127 (25.2)	
Informal income generating activities	349 (69.7)	323 (64.1)	
Formal income generating activities	24 (4.8)	17 (3.4)	
Marital status *n* (%)			1,005
Married—Monogamous	344 (67.7)	348 (69.1)	
Married—Polygamous	28 (5.6)	40 (7.9)	
Living with partner, not married	17 (3.4)	6 (1.2)	
Single	66 (13.2)	62 (12.3)	
Widowed	8 (1.6)	4 (0.8)	
Divorced/separated	38 (7.6)	44 (8.7)	
Household size, mean ± SD	6.8 ± 0.14	6.9 ± 0.12	1,003
Household food expenditure per person per day (TSh), mean ± SD^a^	7,633 ± 4,384	7,974 ± 5,652	935
Worry about enough food in past 4 weeks *n* (%)			1,003
Never	307 (61.4)	310 (61.6)	
Rarely	113 (22.6)	123 (24.5)	
Sometimes	30 (6.0)	21 (4.2)	
Often	50 (10.0)	49 (9.7)	
BMI (kg/m^2^), mean ± SD	24.4 ± 6.0	24.2 ± 5.1	989
Underweight (BMI < 18.5 kg/m^2^), *n* (%)	39 (7.9)	39 (7.9)	
Overweight (BMI > 25 kg/m^2^), *n* (%)	171 (34.5)	168 (34.1)	

Abbreviations: BMI, body mass index; CON, control; INT, intervention.

^a^ Exchange rate in November 2016: 2,200 Tanzanian shillings per $US1.

To understand the implications of these three analytic strategies, we report results from (a) unadjusted regressions with fixed effect for matched pair (the design‐based estimator), (b) weighted regressions with inverse probability of treatment weights and (c) weighted regressions with combined treatment‐and‐censoring weights. We focus the reporting and interpretation on the final (c) model results but present the unadjusted and inverse probability of treatment weighted estimates in Tables 3 and 4.

To evaluate effects of the intervention on women's dietary diversity scores and household food insecurity scores, we fit linear regression models with fixed effects for matched‐village pair and inverse probability of treatment and censoring weights with robust variance estimators (Thoemmes and Ong, 2016). We computed inverse probability of treatment weights by predicting probabilities of being treated from logistic regression models stratified by matched pair controlling for baseline response variable, baseline wealth quintiles, baseline education level and baseline livestock ownership. Censoring weights were similarly obtained from pair‐stratified logistic regression models that included any significant predictors of loss to follow up: baseline marital status, baseline livestock ownership and baseline wealth quintiles (supporting information). To evaluate the effects of the intervention on the dichotomous outcomes of (a) achieving MDD‐W, (b) consuming a given food or (c) having a certain level of food insecurity, we fit linear probability models such that the intervention effect is expressed as a risk difference (RD). We do not report risk ratios, as there was a noticeable amount of effect heterogeneity across village pairs and a summary risk ratio would be misleading.

We control for the family‐wise error rate—the probability of getting at least one false positive result—for multiple comparisons using the Bonferroni correction. We performed 16 hypothesis tests and therefore set our critical *p* value (probability level, *p* value) to 0.003. Missing data among surveyed households were less than 5%; therefore, we performed a complete‐case analysis.

Models that account for pair‐matched cluster‐randomized design estimate the effect while assuming that observations within a pair are independent (Imai et al., 2009). To evaluate potential additional within‐village correlation in outcomes within pair, we fit mixed‐effects models with matched pair dummy variables and village‐level random effects and estimated an intraclass correlation coefficient (ICC) for dietary diversity scores of 0.004. From this, we conclude that additional within‐village clustering is minimal, and we do not further adjust for clustering beyond reporting Huber–White robust standard errors. We also estimated and report bootstrapped confidence intervals using wild bootstrap (null imposed, 999 replications, Wald test and Rademacher weights) and find similar values.

### 2.5 Ethical considerations

The study protocol was approved by the institutional review boards of Ifakara Health Institute, the National Institute for Medical Research of Tanzania (NIMR/HQ/R. 8 a/Vol. IX/2262) and the Harvard T.H. Chan School of Public Health.

## 3 RESULTS

In total, 1,006 participants were randomized into 504 INT households and 502 CON households. After loss to follow up, 876 participants remained for analysis of the primary and secondary outcomes, 454 in INT and 422 in CON. At baseline, INT and CON differed statistically in wealth quintiles, dietary diversity and number of participants who have no income‐generating activities (Tables 1 and 2). The average household size was 6.8, and the average participant was 32 years of age. More than two thirds of the participating women were engaged in informal income‐generating activities, and the same proportions were married monogamously and had completed some education (Table 1). On average, CON participants consumed 2.3 food groups per day at baseline compared with 3.1 food groups among INT participants (*p* < 0.001; Table 2).

**TABLE 2:** Baseline unadjusted proportions of mothers that consumed a given food group in the past 30 days

Variable	CON	INT	∂ %
Dietary diversity score, mean ± SD	2.3 ± 1.1	3.1 ± 1.6	
Dietary diversity score ≥5, *n* (%)	14 (2.8)	91 (18.2)	15.4
Household food insecurity score, mean ± SD	5.8 ± 8.0	5.3 ± 7.8	
Mild or no food insecurity, *n* (%)	258 (51.6)	267 (53.1)	1.5
Moderate food insecurity, *n* (%)	41 (8.2)	58 (11.5)	4.3
Severe food insecurity, *n* (%)	201 (40.0)	178 (35.3)	−4.7
Consumption of individual food groups, *n* (%)			
Starches	499 (99.4)	499 (99.4)	0.0
Nuts and seeds	0 (100.0)	3 (0.6)	0.6
Beans and peas	11 (2.2)	40 (8.0)	5.8
Dairy	14 (2.8)	46 (9.2)	6.4
Flesh foods	290 (57.8)	398 (79.3)	21.5
Eggs	1 (0.2)	4 (0.8)	0.6
Dark ‐green vitamin A‐rich vegetables	129 (25.7)	142 (28.3)	2.6
Other vitamin A rich vegetables and fruits	32 (6.4)	63 (12.6)	6.2
Other vegetables	123 (24.5)	224 (44.6)	20.1
Other fruits	34 (6.8)	136 (27.1)	20.3
Household cultivated vegetables, *n* (%)	8 (1.6)	44 (8.7)	7.1
Household cultivated roots or legumes, *n* (%)	98 (19.5)	56 (11.1)	−8.4
Number of crops grown, mean ± SD	1.6 ± 1.6	2.4 ± 2.0	

Abbreviations: CON, control; INT, intervention.

### 3.1 Impact on dietary diversity

**FIGURE 1:**
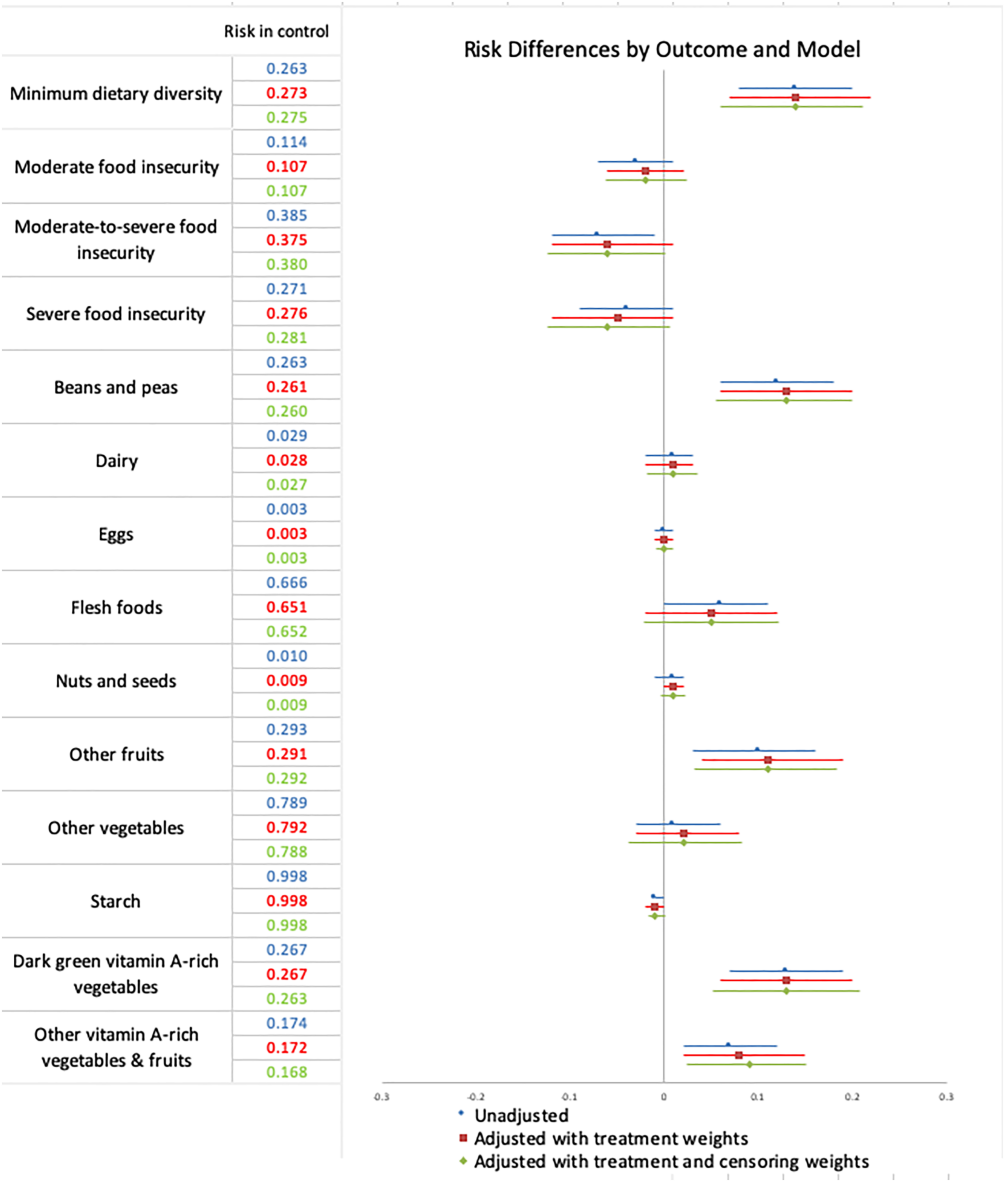
Risk differences between intervention (INT) and control (CON) households after 12 months of follow up. Adjusted models show difference in risk of outcome expressed as a probability of outcome in INT compared with CON group

At 12 months, after adjusting for baseline covariate imbalances and differential loss to follow up, women in INT consumed an average of 0.50 (95% CI [0.20, 0.80], *p* = 0.001) more food groups per day than women in CON (Table 3). Additionally, among women in INT, the proportion of women consuming at least five food groups per day was 14 percentage points higher than that of women in CON (95% CI [6, 22], *p* = 0.001), after adjusting for baseline differences and loss to follow up (Table 4; Figure 1).

**TABLE 3:** Differences in dietary diversity score and household food insecurity score between intervention (INT) and control (CON) after 12 months of follow up

	Unadjusted					Adjusted with treatment weights					Adjusted with treatment and censoring weights						
Outcome	*β*1	*N*	LCI	UCI	*p*	*β*1	*N*	LCI	UCI	*p*	*β*1	*N*	LCI	UCI	*p*	Bootstrapped confidence interval	
Dietary diversity score	0.53	873	0.33	0.74	<0.001	0.50	865	0.20	0.80	0.001	0.50	862	0.20	0.80	0.001	0.19	0.81
Household food insecurity access scale	−0.39	874	−1.14	0.37	0.312	−0.53	862	−1.46	0.41	0.272	−0.58	859	−1.50	0.35	0.222	−1.51	0.36
Number of crops grown	3.04	874	2.79	3.31	<0.001	2.65	870	2.35	2.96	<0.001	2.66	867	2.35	2.96	<0.001	2.34	2.98

*Note:* Adjusted models show risk differences from linear models with fixed effects for matched village pair, inverse probability of treatment and censoring weights, and robust standard errors. Confounders adjusted for by treatment weights include baseline response variable, baseline wealth quintiles, baseline education level and baseline livestock ownership. The Bonferroni corrected critical *p* value is 0.003. Interpretations: *β*1, effect estimate.

Abbreviations: LCI, lower bound for 95% confidence interval; SE, standard error; UCI, upper bound for 95% confidence interval.

**TABLE 4:** Risk differences between intervention (INT) and CON after 12 months of follow up

	Unadjusted						Adjusted with treatment weights						Adjusted with treatment and censoring weights							
Outcome	RD	Risk in CON^a^	*N*	LCI	UCI	*p*	RD	Risk in CON^a^	*N*	LCI	UCI	*p*	RD	Risk in CON^a^	*N*	LCI	UCI	*p*	Bootstrapped 95% confidence interval	
Minimum dietary diversity	0.14	0.26	876	0.08	0.20	<0.001	0.14	0.27	868	0.07	0.22	<0.001	0.14	0.27	865	0.06	0.22	0.001	0.06	0.21
Moderate‐to‐severe food insecurity	−0.07	0.38	874	−0.12	−0.01	0.017	−0.06	0.38	866	−0.12	0.01	0.078	−0.06	0.38	863	−0.13	0.00	0.059	−0.12	0.00
Severe food insecurity	−0.04	0.27	874	−0.09	0.01	0.156	−0.05	0.28	866	−0.12	0.01	0.096	−0.06	0.28	863	−0.12	0.00	0.065	−0.12	0.01
Beans and peas	0.12	0.26	876	0.06	0.18	<0.001	0.13	0.26	864	0.06	0.20	<0.001	0.13	0.26	861	0.06	0.19	<0.001	0.06	0.20
Dairy	0.01	0.03	876	−0.02	0.03	0.501	0.01	0.03	871	−0.02	0.03	0.570	0.01	0.03	868	−0.02	0.04	0.532	−0.02	0.04
Eggs	0.00	0.00	876	−0.01	0.01	0.651	0.00	0.00	869	−0.01	0.01	0.923	0.00	0.00	866	−0.01	0.01	0.961	−0.01	0.01
Flesh foods	0.06	0.67	876	0.00	0.11	0.041	0.05	0.65	871	−0.02	0.12	0.138	0.05	0.65	868	−0.02	0.12	0.164	−0.02	0.12
Nuts and seeds	0.01	0.01	876	−0.01	0.02	0.282	0.01	0.01	868	0.00	0.02	0.187	0.01	0.01	865	0.00	0.02	0.205	0.00	0.02
Other fruits	0.10	0.29	876	0.03	0.16	0.002	0.11	0.29	871	0.04	0.19	0.004	0.11	0.29	868	0.03	0.18	0.005	0.03	0.18
Other vegetables	0.01	0.79	876	−0.03	0.06	0.556	0.02	0.79	866	−0.03	0.08	0.402	0.02	0.79	863	−0.04	0.08	0.448	−0.04	0.08
Starch	−0.01	1.00	876	−0.01	0.00	0.083	−0.01	1.00	867	−0.02	0.00	0.093	−0.01	1.00	864	−0.02	0.00	0.094	−0.02	0.00
Dark Green vitamin A‐rich vegetables	0.13	0.27	876	0.07	0.19	<0.001	0.13	0.27	870	0.06	0.20	0.001	0.13	0.26	867	0.06	0.20	<0.001	0.05	0.21
Other vitamin A‐rich vegetables and fruits	0.07	0.17	876	0.02	0.12	0.007	0.08	0.17	871	0.02	0.15	0.007	0.09	0.17	868	0.03	0.15	0.005	0.02	0.15

*Note:* Adjusted models show risk differences from linear regression models with fixed effects for matched village pair, inverse probability of treatment and censoring weights, and robust standard errors. Confounders adjusted for by treatment weights include baseline response variable, baseline wealth quintiles, baseline education level and baseline livestock ownership. The Bonferroni corrected critical *p* value is 0.003.

Abbreviations: CON, control; LCI, lower bound for 95% confidence interval; RD, risk difference; SE, standard error; UCI, upper bound for 95% confidence interval.

^a^ Risk of the outcome in control, averaged over the pairs.

In fully adjusted models, women in INT were also more likely to consume vitamin A‐rich dark green vegetables (RD = 13% pt. increase in probability of outcome, 95% CI [6, 20], *p* < 0.001), other vitamin A‐rich vegetables and fruits (RD = 9% pt., 95% CI [3, 15], *p* = 0.005) or beans and peas (RD = 13% pt., 95% CI [6, 19], *p* < 0.001) compared with women in CON (Table 4; Figure 1). After the Bonferroni correction using critical *p* value for significance = 0.003, the difference in probability of consuming other vitamin A‐rich vegetables and fruits was not statistically significant (*p* value = 0.005), but consumption of dark green vegetables and consumption of beans and peas remained significant.

### 3.2 Impact on household food security

In fully adjusted models, the household food insecurity score of women in INT was not significantly different (*β* = −0.58; 95% CI [−1.50, 0.35], *p* = 0.222) compared with CON (Table 3). However, the probability of SFI among INT was 6 percentage points (95% CI [−13, 0], *p* = 0.059) lower compared with CON (Table 4). The probability of moderate‐to‐severe food insecurity was also 6 percentage points (95% CI [−12, 0], *p* = 0.065) lower in INT compared with CON (Table 4). The difference in probability of moderate food insecurity between INT and CON households was not statistically significant (RD = −2% pt., 95% CI [−6, 2], *p* = 0.371) (Table 4).

## 4 DISCUSSION

We found positive effects on women's dietary diversity among participants receiving the HFP intervention. Our analysis also confirms that integrated nutrition and agricultural interventions can increase consumption of nutrient rich foods. Additionally, households in intervention (INT) villages were marginally less likely to experience moderate‐to‐severe food insecurity compared with households in control (CON) villages.

Our results are consistent with previous studies that found significant effects of HFP on individual food groups. A randomized controlled trial of an integrated HFP intervention in Burkina Faso found significant effects on fruit consumption and a marginally significant improvement both in consumption of meat or poultry and in dietary diversity. (Olney et al., 2016) Our results support these findings: the largest impact was seen on dark green vitamin A‐rich vegetables and beans and peas, and smaller, marginally significant impacts were seen on consumption of other fruits and flesh foods. However, our study also found that the participants in INT consumed more food groups overall than participants from CON and their probability of consuming a minimally diverse diet (consisting of five or more food groups out of 10 per day) was higher after 12 months of the intervention. Participants in intervention villages grew on average more crops that corresponded to the seeds provided, than those in control villages, indicating a plausible pathway through which consumption of dark green vitamin A‐rich vegetables increased (supporting information). Participants also increased their consumption of beans and peas, other fruits and other food groups. We did not facilitate production for these crops and hypothesize that the increased consumption is indicative of behavioural change as a result of the nutrition education provided during the programme. Additionally, INT women may have increased their disposable food budget by spending less on the crops now grown and thus have been more likely to afford other foods not grown at the household.

Women's dietary diversity and household food security have been linked to positive health and nutritional outcomes both among women themselves and their children. In several studies, a positive association was found between dietary diversity and micronutrient adequacy (Arsenault et al., 2013; Henjum et al., 2015), dietary quality and nutritional status (Burney, Alaofe, Naylor and Taren, 2016), and vitamin A status (Fujita, Lo and Baranski, 2012) of women across multiple contexts. Women's dietary diversity has also been associated with higher child dietary diversity, suggesting positive effects beyond that of the participant alone (Amugsi, Mittelmark and Oduro, 2015). Maternal nutrient intake has also been associated with decreased risk of preterm birth (Zhang, Zhou, Perkins, Wang and Sun, 2017). Although we find effects on diet after 12 months, effects on nutritional status outcomes may take longer to establish.

Previous cross‐sectional results from our study showed an association between crop diversity, production of pulses and production of vegetables with women's dietary diversity score (Bellows et al., 2019). Additionally, our previous research found positive associations between the gardening activities of one household and the dietary diversity of neighbouring households, suggesting that there may be positive externalities of the intervention to the larger community, and not just the beneficiary households. Specifically, women who lived near a neighbour who grew crops in a home garden had 0.53 higher dietary diversity score (consumed 0.53 more food groups per day, on average) and had 2.77 times the odds of achieving minimum dietary diversity compared with those who did not (Blakstad et al., 2019). The magnitude of impact on neighbours' dietary diversity is of similar magnitude as the effect size of INT on study participants' dietary diversity (0.50 food groups). Our focus group data suggest that neighbours could increase their dietary diversity both through growing crops themselves and through receiving vegetables directly from women growing crops. By comparison, women in INT may either consume crops from their garden or spend their food budget differently as a result of the nutrition education and changing diet preferences.

Although the impact on food insecurity was not statistically significant, we observed a trend in the reduction of the prevalence of household SFI. This trend is consistent with previous quasi‐experimental studies evaluating the effect of HFP programmes on household food security (Bushamuka et al., 2005; Talukder et al., 2000). Food insecurity has been positively associated with undernutrition among children, adolescents and women (Bukania et al., 2014; Cordeiro, Wilde, Semu and James Levinson, 2012; McDonald et al., 2015). Improvements in dietary diversity and food security through agricultural interventions may improve nutrition, health and economic outcomes among women and their children. In rural areas with high prevalence of poverty, HFP programmes and activities could not only improve dietary quality but also serve as a buffer against high household SFI. This may be particularly important during periods in which market food prices are high, for instance, during the dry season or planting season. Our study did not capture differences in diet or food security across seasons, as data collection occurred in the same (nonharvesting) month for baseline and at the 12‐month follow up. Therefore, we cannot make claims about the impact on food security during harvesting seasons, when the impact could be greater.

Our study has certain limitations to note. First, the study was randomized within pairs at the cluster level. Balance in baseline characteristics is not guaranteed when the number of units of randomization is small. Despite the pairwise matching and intervention randomization of the study design, we still observed imbalances in baseline characteristics. We accounted for confounding from covariate imbalances and for the similarities of observations within a cluster (village) by using an inverse probability of intervention weights. Additionally, we noted a moderately high level of attrition that differed between study arms, with a slightly higher rate of attrition in the intervention group. It is possible that this attrition is related to the time‐intensive nature of the intervention. This may raise concerns of potential unintended consequences of the impact of intensive agriculture approaches on time allocation of households particularly for women. Dietary diversity captured usual intake over the past 30 days and household food insecurity was estimated for the past 4 weeks and is therefore unable to capture the long‐term stability and diversity of the food supply. Lastly, dietary diversity scores and the MDD‐W indicator are usually derived from diet intake data collected by 24‐h recall surveys. Our study uses 30‐day FFQs to better capture long‐term usual intake, and therefore, our dietary diversity scores may not be directly comparable with dietary diversity scores calculated using 24‐h recall.

Despite baseline imbalances, our study is a randomized trial with a statistically rigorous analysis, and therefore, internal validity is expected to be high. Our threshold for rejecting the null hypothesis of no effect is conservative after applying the Bonferroni correction for our critical *p* value. Additionally, as the demographic characteristics of the study were representative of broader rural Tanzania, we anticipate that our results may be applicable to other similar regions of Tanzania where dietary diversity is low (Huang et al., 2018). Because agricultural productivity and dietary diversity could be enhanced among our participants, similar improvements could be feasible for other rural areas in Tanzania and East Africa.

The intervention package also shows promise for scale‐up. Currently, AEWs are employed by the Tanzanian government to train and support local farmers to increase productivity. CHWs are another existing cadre of health professionals in rural Tanzania. Typically, CHWs and AEWs are siloed, even though both operate at the village level and serve the same population. Through enhanced training, these existing cadres of health workers developed the capacity to provide households with an understanding and appreciation of agriculture‐for‐nutrition concepts and nutritional knowledge and public health topics that are not covered in the routine training curricula. Our findings suggest that developing coordinated multisectoral approaches to integrating these two cadres is feasible, and likely to have significant benefits for addressing malnutrition. It is plausible that the intervention may have cost households a substantial amount of labour time when planting and weeding to yield vegetables a few months later; hence, future studies need to assess the relative costs and cost‐effectiveness of this intervention.

Our trial is among the first cluster‐randomized controlled trials to find significant impacts on dietary diversity scores and probability of consuming five or more food groups per day. The positive impacts of the programme on food security, on consumption of individual food groups, on the odds of consuming a minimally diverse diet and on the average number of food groups consumed are promising. The results were achieved over a relatively short period of 12 months, and future research should examine whether the effects are stronger, smaller or maintained in the long run. Additionally, because studies have shown links between dietary diversity and serum haemoglobin and nutritional status in both children and adults, future trials should assess the direct effect of nutrition‐sensitive interventions on functional nutritional outcomes that are likely to be affected by such interventions (Korkalo et al., 2017; McDonald et al., 2015). Our earlier finding that neighbouring households in intervention villages are more likely to grow a home garden indicates that the practice could have spillover benefits beyond the intended households. Together, these aspects suggest that home gardening programmes could help foster wide‐reaching nutrition and health gains in rural farming communities.

## CONFLICTS OF INTEREST

All authors declare no conflicts of interest.

## CONTRIBUTIONS

MMB, DM, ALB, CRC, WWF, JK, RAN and HM designed the research study; DM, KM, HM and WF conducted field research and oversaw implementation; MB and JTC analysed the data; MB wrote the paper; MMB and WWF have primary responsibility for final content. ALB, CRC and WWF critically revised the manuscript for important intellectual content. All authors have read and approved the final manuscript and are accountable for all aspects of the work.

## CLINICAL TRIAL REGISTRATION

ClinicalTrials.gov NCT03311698.

## Supporting information


**Table S1:** Food Groups Included in Dietary Diversity Score
Supplementary Table S2: Baseline characteristics by loss‐to‐follow‐up status.
Supplementary Table S3: Differences in household growing crops at 12 months
**Figure S1:** CONSORT flow chart of enrollment, intervention allocation, follow‐up, and data analysis in the cluster‐randomized trial.
