# Supplementary material for: Home gardening improves dietary diversity, a cluster‐randomized controlled trial among Tanzanian women
Source: Matern Child Nutr. 2020 Nov 26;17(2):e13096. doi: 10.1111/mcn.13096 (PMC7988851; doi:10.1111/mcn.13096)
Supplement: Supplementary file 1 — Table S1: Food Groups Included in Dietary Diversity Score Supplementary Table S2: Baseline characteristics by loss‐to‐follow‐up status. Supplementary Table S3: Differences in household growing crops at 12 months Figure S1: CONSORT flow chart of enrollment, intervention allocation, follow‐up, and data analysis in the cluster‐randomized trial. [file MCN-17-e13096-s001.docx]

**Supplementary table 1: Food Groups Included in Dietary Diversity Score**

| **Food Group** | **Food item** |
| --- | --- |
| Starchy Staples | Porridge, ugali (stiff porridge), bread, spaghetti, chapati, donut, rice cake, biscuit, cake, maize, rice, Irish potato, cassava, sweet potato, taro |
| Flesh Foods | Beef, goat, pork, chicken, fish |
| Vitamin A Dark Green Vegetables | Spinach, cassava leaves, sweet potato leaves, amaranth greens, pumpkin leaves, cowpea leaves, Chinese cabbage |
| Other Vegetables | Lettuce, eggplant, cucumber, cabbage, green pepper, okra, bitter tomato |
| Other Fruits | Ripe banana, tamarind, plum, tangerine, lemon/lime, jackfruit, baobab, guava, watermelon, peaches, avocado, pineapple, pineapple juice, orange, orange juice, passion juice |
| Other Vitamin A Vegetables & Fruits | Mango, papaya, pumpkin, carrot, sweet potato |
| Dairy | Cow's milk, tea with milk, coffee with milk, ice cream |
| Beans & Peas | Kidney beans, green mung beans, pigeon peas, chickpeas, cow peas, green peas |
| Eggs | Eggs |
| Nuts & Seeds | Bambara nuts, ground nuts |

Adapted with permission from Bellows et al., 2019.

**Supplementary table 2: Baseline characteristics by loss-to-follow-up status.**

| **Characteristic** | **Retained** | **Lost to follow-up** |
| --- | --- | --- |
| Treatment n (%) | 49 (37.7) | 455 (51.9) |
| Livestock ownership n (%) | 289 (33.0) | 21 (16.2) |
| Wealth Quintile Score n (%) |  | |
| First (Lowest) | 177 (20.2) | 15 (11.5) |
| Second | 148 (16.9) | 33 (25.4) |
| Third | 203 (23.2) | 29 (22.3) |
| Fourth | 166 (19.0) | 24 (18.5) |
| Fifth (Highest) | 171 (19.5) | 28 (21.5) |
| Education n (%) |  | |
| No education | 284 (32.5) | 44 (33.9) |
| Primary | 510 (58.3) | 68 (52.3) |
| Secondary | 74 (8.5) | 17 (13.1) |
| Higher Education (high school or higher) | 7 (0.8) | 1 (0.8) |
| Type of Employment n (%) |  | |
| No Income Generating Activity | 190 (21.7) | 24 (18.5) |
| Informal Income Generating Activities | 591 (67.5) | 81 (62.3) |
| Formal Income Generating Activities | 32 (3.7) | 9 (6.7) |
| Marital Status n (%) |  | |
| Married – monogamous | 614 (70.2) | 78 (60.0) |
| Married – polygamous | 65 (7.4) | 3 (2.3) |
| Living with partner, not married | 17 (1.9) | 6 (4.6) |
| Single | 101 (11.5) | 27 (20.8) |
| Widowed 6 | 11 (1.3) | 1 (0.8) |
| Divorced/separated 7 | 67 (7.7) | 15 (11.5) |
| Household size | 6.9 ± 2.7 | 6.5 ± 3.1 |
| Household food expenditure per person per day (TSh) mean (SD) ^1^ | 7855 ± 5166 | 7471 ± 4188 |
| Worry about enough food in past 4 weeks n % |  | |
| Never | 535 (61.3) | 82 (63.1) |
| Rarely | 213 (24.4) | 23 (17.7) |
| Sometimes | 40 (4.6) | 11 (8.5) |
| Often | 85 (9.7) | 14 (10.8) |
| BMI, kg/m^2^ | 24.5 ± 5.6 | 23.3 ± 4.8 |
| Underweight (BMI <18.5 kg/m^2^), n % | 67 (7.8) | 11 (8.5) |
| Overweight (BMI >25 kg/m^2^), n % | 305 (35.5) | 34 (26.36) |

Differences in baseline characteristics between households retained vs lost to follow-up.

**Supplementary table 3: Differences in household growing crops at 12 months**

|  | **Unadjusted proportions of household growing crops at 12 months** | | **Adjusted with treatment and censoring weights** | | | | |
| --- | --- | --- | --- | --- | --- | --- | --- |
| **Outcome** | **CON**  N (%) | **INT**  N (%) | RD | Risk in CON^1^ | LCI | UCI | p |
| Amaranthus | 0 (0.0) | 46 (10.2) | 0.09 | -0.01 | 0.07 | 0.12 | <0.001 |
| Eggplant | 1 (0.1) | 37 (8.2) | 0.07 | 0.00 | 0.05 | 0.10 | <0.001 |
| Spinach | 0 (0.0) | 188 (41.5) | 0.42 | 0.00 | 0.38 | 0.47 | <0.001 |
| Tomato | 8 (1.9) | 123 (27.2) | 0.23 | 0.00 | 0.19 | 0.28 | <0.001 |
| Okra | 24 (5.7) | 215 (47.5) | 0.38 | 0.06 | 0.32 | 0.44 | <0.001 |
| Chinese Cabbage | 0 (0.0) | 92 (20.3) | 0.19 | -0.02 | 0.15 | 0.23 | <0.001 |

Adjusted proportions of households in INT and CON that grew crops corresponding to seeds provided through the intervention. Adjusted models show risk differences from linear regression models with fixed effects for matched village pair, inverse probability of treatment and censoring weights, and robust standard errors. Confounders adjusted for by treatment weights include baseline response variable, baseline wealth quintiles, baseline education level, and baseline livestock ownership. The Bonferroni corrected critical p-value is 0.003. Interpretations: SE= standard error, LCI/UCI= lower/upper bound for 95% confidence interval. 1: Risk of the outcome in control, averaged over the pairs.
